# Metabolic Engineering of *Fusarium oxysporum* to Improve Its Ethanol-Producing Capability

**DOI:** 10.3389/fmicb.2016.00632

**Published:** 2016-05-04

**Authors:** George E. Anasontzis, Elisavet Kourtoglou, Silas G. Villas-Boâs, Dimitris G. Hatzinikolaou, Paul Christakopoulos

**Affiliations:** ^1^Microbial Biotechnology Unit, Sector of Botany, Department of Biology, National and Kapodistrian University of AthensZografou, Greece; ^2^BIOtechMASS Unit, Biotechnology Laboratory, School of Chemical Engineering, National Technical University of AthensZografou, Greece; ^3^Centre for Microbial Innovation, School of Biological Sciences, University of AucklandAuckland, New Zealand; ^4^Biochemical and Chemical Process Engineering, Division of Sustainable Process Engineering, Department of Civil, Environmental and Natural Resources Engineering, Luleå University of TechnologyLuleå, Sweden

**Keywords:** *Fusarium oxysporum*, ethanol, consolidated bioprocessing, filamentous fungi, biofuels, fermentation, γ-aminobutyric acid shunt, NADPH regeneration

## Abstract

*Fusarium oxysporum* is one of the few filamentous fungi capable of fermenting ethanol directly from plant cell wall biomass. It has the enzymatic toolbox necessary to break down biomass to its monosaccharides and, under anaerobic and microaerobic conditions, ferments them to ethanol. Although these traits could enable its use in consolidated processes and thus bypass some of the bottlenecks encountered in ethanol production from lignocellulosic material when *Saccharomyces cerevisiae* is used—namely its inability to degrade lignocellulose and to consume pentoses—two major disadvantages of *F. oxysporum* compared to the yeast—its low growth rate and low ethanol productivity—hinder the further development of this process. We had previously identified phosphoglucomutase and transaldolase, two major enzymes of glucose catabolism and the pentose phosphate pathway, as possible bottlenecks in the metabolism of the fungus and we had reported the effect of their constitutive production on the growth characteristics of the fungus. In this study, we investigated the effect of their constitutive production on ethanol productivity under anaerobic conditions. We report an increase in ethanol yield and a concomitant decrease in acetic acid production. Metabolomics analysis revealed that the genetic modifications applied did not simply accelerate the metabolic rate of the microorganism; they also affected the relative concentrations of the various metabolites suggesting an increased channeling toward the chorismate pathway, an activation of the γ-aminobutyric acid shunt, and an excess in NADPH regeneration.

## Introduction

Lignocellulose is the complex matrix of cellulose, hemicellulose, and lignin that constitutes the plant cell wall. Being a waste product from agricultural activities, forestry, and the pulp and paper industry, lignocellulose can be a sustainable source of carbohydrates for biotechnological applications (Fujita et al., 2002; Malherbe and Cloete, 2002).

The commonly perceived process for lignocellulose use in a biorefinery for ethanol production comprises of two main steps; hydrolysis and fermentation. Hydrolysis refers to the breakdown of polysaccharides present in biomass to monosaccharides and fermentation refers to the use of these monosaccharides for the synthesis of the end-product. Hydrolysis and fermentation can be performed either sequentially or in parallel (Ask et al., 2012). Selection of the set-up depends on the raw material. However, since each set-up has different requirements in terms of physical and chemical conditions, different biomass-degrading enzyme mixtures are required for the efficient release of the available sugars. For example, when hydrolysis and fermentation are performed simultaneously, the temperature is maintained at 30°C, which is not necessarily the optimum temperature for the commercial enzyme mixtures. When hydrolysis is performed separately from fermentation, the temperature can be closer to the optimum for the enzyme mixture, but other factors—such as product inhibition—can affect the efficiency of the process (Ask et al., 2012).

As far as the fermentation part is concerned *Saccharomyces cerevisiae* is undoubtedly the most important microorganism, due to its high productivity (reported as high as 3.3 g_ethanol_ L^−1^ h^−1^; Cavka et al., 2010) and generally high metabolic rate, which make it a suitable microorganism for industrial use (Kuyper et al., 2004). This microorganism is capable of fermenting hexoses, such as glucose and mannose, to ethanol. Glucose is present in various concentrations and structures in plant biomass, but predominantly in the form of cellulose; mannose is abundant as a hemicellulose constituent from gymnosperms. The capacity of yeast to efficiently produce ethanol has prompted its use for the production of biofuels, and as a production host for the biosynthesis of other bulk and fine biochemicals (Nevoigt, 2008).

Despite its obvious advantages, *S. cerevisiae* has several drawbacks that hinder its use in lignocellulose-based processes; these include its dependence on previous chemical or enzymatic steps in order to break down the plant cell wall biomass to monosaccharides, and also its inability to metabolize pentoses such as xylose (la Grange et al., 2010). Despite the development of *S. cerevisiae* strains capable of fermenting xylose (Bettiga et al., 2009), which has allowed extensive work on increasing tolerance to inhibitors present in lignocellulose (Koppram et al., 2012) and on optimizing the fermentation process from lignocellulosic materials (Koppram et al., 2013), these bottlenecks have also encouraged the development of microorganisms that can use plant biomass, saccharify the polymers, and ferment the product of interest in a consolidated bioprocess (Lynd et al., 1996). This can be based either on the engineering of microorganisms that are naturally capable of breaking down biomass in order to improve the efficiency of the production step, or on the engineering of non-cellulolytic microorganisms, but with a high yield for the product of interest, in order to acquire the ability to extract the sugars from lignocellulosic biomass (la Grange et al., 2010). Despite the progress achieved in both of these strategies, outlined in recent reviews (la Grange et al., 2010; den Haan et al., 2015), there are no such strains available today that could make the consolidated bioprocessing system advantageous on an industrial scale.

*Fusarium oxysporum* is a filamentous fungus. It is one of the few fungal species reported to ferment plant carbohydrate polymers to ethanol in a one-step process (Christakopoulos et al., 1989; Singh and Kumar, 1991; Anasontzis et al., 2011). It can produce a range of plant cell wall degrading enzymes (Christakopoulos et al., 1996a,c, 1997) and ferment D-glucose, D-xylose, cellulose, and hemicellulose to ethanol. Compared to yeasts, its main disadvantage is the low conversion rate (Singh and Kumar, 1991).

The strain F3 of *F. oxysporum* was selected for its high cellulase production, and it has been extensively studied (Christakopoulos et al., 1989; Panagiotou et al., 2005a,b,c, 2008). The accumulation by *F. oxysporum* F3 of high levels of glucose-1,6-phosphate at the end of both the aerobic and the anaerobic phases, when glucose was used as carbon source, was attributed to what was believed to be a low phosphoglucomutase activity, which was consequently restricting the metabolism of glucose toward cell wall biosynthesis (Panagiotou et al., 2005c), glycolysis, and ethanol production. This enabled us to study this enzyme further and to show that it follows the ping-pong mechanism for the conversion of glucose-1-P to glucose-6-P and vice versa (Kourtoglou et al., 2011). Also, the accumulation of sedoheptulose-7-P when the microorganism was grown in glucose-xylose mixtures under oxygen-limiting conditions, and of erythrose-4-P during xylose consumption (Panagiotou et al., 2005b), indicated a second bottleneck in the *F. oxysporum* metabolism. Fan et al. reported the effect on *F. oxysporum* of the overexpression of transaldolase genes from *Pichia stipitis* and *S. cerevisiae*, which increased both xylose consumption and ethanol production when either glucose or xylose was used as carbon source (Fan et al., 2011a,b) in shake flask batch cultures.

We had previously investigated the effect of overproduction of these two enzymes on the metabolism of *F. oxysporum* under aerobic conditions. Through a double transformation using customized vectors providing hygromycin resistance, we had generated a number of strains that constitutively overexpressed the phosphoglucomutase and/or the transaldolase gene under the regulation of the *gpd*A promoter of *Aspergillus nidulans*. Among these strains, FF11 produced the highest amounts of both enzymes. Thus, we compared FF11 and the parental strain F3 in order to evaluate the effect of the modification on the growth characteristics of the microorganism. We found that the applied modification increased the growth rate and the fungal biomass yield when glucose was used as carbon source. We also detected changes in the metabolome that indicated a facilitation of the channeling of the sugars through the glycolysis and pentose phosphate metabolism, such as the accumulation of glutamate as a possible response to the high NADPH availability from higher flux through the pentose phosphate pathway (Anasontzis et al., 2014a).

In this study, we evaluated the effect of the constitutive production of phosphoglucomutase and tranlsaldolase on ethanol productivity when glucose was used as carbon source. Thus, we compared the parental strain F3 with the genetically modified FF11 under anaerobic conditions. We showed that the latter outperformed F3 in terms of ethanol production, glucose consumption and by-product generation. We also used metabolomics tools, in an attempt to understand if the metabolome profile indicates a continuously high NADPH and NADH availability which resulted in improved fermentation characteristics.

## Materials and methods

### Microorganisms and media

*Fusarium oxysporum* wild type F3 (Christakopoulos et al., 1989) was maintained on PDA plates (Potato Dextrose Agar; Applichem, Germany) and recombinant FF11 (Anasontzis et al., 2014a) was maintained on PDA plates supplemented with 50 mg L^−1^ hygromycin at 4°C, following growth for 4 days at 25°C.

The basal medium for growth in bioreactors and for metabolome analysis had the following composition (in g^.^L^−1^): NaH_2_PO_4_.2H_2_O, 7; Na_2_HPO_4_.2H_2_O, 9.5; KH_2_PO_4_, 1; (NH_4_)_2_HPO_4_, 10; MgSO_4_.7H_2_O, 0.3; CaCl_2_.2H_2_O, 0.3 (Christakopoulos et al., 1996b).

Precultures were grown for 24 h in 500-mL Erlenmeyer flasks (100 mL working volume) using the basal medium supplemented with glucose at 20 g^.^L^−1^ as carbon source. The whole preculture was used to inoculate a 2-L stirred tank bioreactor (BioFlo310, Benchtop Fermenter/Bioreactor; New Brunswick Scientific GmbH, Germany) with a working volume of 1.5 L and at a final concentration of 7% (v/v), under aerobic conditions as previously described (Anasontzis et al., 2014a). After the end of the aerobic phase, which corresponded to glucose depletion, the air supply was stopped and 60 g of sterilized glucose was added.

### Genetic modification

Genetic modification of *F. oxysporum* F3 was performed as previously described (Kourtoglou et al., 2011; Anasontzis et al., 2014a). In brief, the genes encoding for translaldolase and phosphoglucomutase—*tal* and *pgm*— were isolated from the genomic DNA of *F. oxysporum* with PCR and appropriately designed primers. The overexpression vectors were constructed by substituting the *xyl*2 gene from pBlXyl (Anasontzis et al., 2011) with the PCR fragments of the two genes. pBLXyl had the *gpd*A promoter upstream and the *trp*C terminator of *A. nidulans* downstream the xyl2. The hygromycin resistance gene was isolated from the plasmid pCSN44 and ligated to the plasmids, after digestion with *Xho*I. The resulting plasmids pBlTAL-hyg (Genebank accession JF756587) and pBLPGM-hyg (Anasontzis et al., 2011; Kourtoglou et al., 2011) were then used for the co-transformation of *F. oxysporum* F3. Protoplast preparation and transformation were performed as previously described (Kourtoglou et al., 2011). Integration of the genes in the genome was confirmed with PCR and Southern analysis, while gene expression was evaluated with northern analysis. The successful overproduction of the two enzymes was verified with enzymatic assays. The transformant FF11, which had incorporated both inserts and overproduced both enzymes, was selected for this study.

### Cell density and glucose concentration

Cell density was calculated based on dry cell weight (DCW). To determine the glucose concentration in the culture medium, samples were first centrifuged at 4°C and 10,000 × g. The supernatant was then analyzed by HPLC, on a Jasco (PU-987) HPLC system equipped with an ion-moderated partition chromatography column (Aminex HPX-87H; Bio-Rad), and a Waters 410 refractive index detector. The mobile phase (5 mM H_2_SO_4_) was 0.6 mL^.^min^−1^ and the temperature 45°C. All measurements are the result of technical duplicates from two identical fermentations (biological duplicates).

### Quenching and sample metabolite analysis

Sample quenching and derivatization, and the subsequent analyses of amino and non-amino organic acids were performed as previously described (Smart et al., 2010; Anasontzis et al., 2014a). In brief, triplicate 50-mL samples from the cultures were obtained at the last sampling point of the anaerobic phase (139.5 h for FF11 and 124 h for F3), either just before depletion of the carbon source or after the culture had shifted to the stationary phase.

The samples were filtered under vacuum to separate the fungal biomass from the culture medium. The mycelium was then washed with saline solution at 4°C and quenched with cold methanol-water (1:1 v/v) at −30°C. From each filtrate, 1 mL was sampled and supplemented with 0.2 μmol 2,3,3,3-d_4_-alanine as internal standard, and stored at −80°C for the extracellular metabolite analysis. From the quenched mycelium, cold methanol-water freeze and thaw cycles quenched the intracellular metabolites. Alanine was also added as internal standard before the extraction. Both quenched culture medium and the extracted intracellular metabolites from the mycelium were freeze-dried using a BenchTop K manifold freeze dryer (VirTis) before chemical derivatization. The derivatization of the freeze-dried samples was performed with the methyl chloroformate method (MCF), and the derivatives were analyzed in an Agilent GC7890 system coupled to a MSD5975 mass selective detector and a ZB-1701 GC capillary column (30 m × 250 μm internal diameter × 0.15 μm with 5-m guard column; Phenomenex) as previously described (Smart et al., 2010). Metabolomic data analysis was performed as previously described (Smart et al., 2010; Anasontzis et al., 2014a). The deconvolution of GC-MS chromatograms and the identification of metabolites was performed with the software AMDIS using an in-house MCF mass spectra library. Both MS spectrum and its respective retention time were used for metabolite identification. The relative abundance of identified metabolites was determined with ChemStation (Agilent) by using the GC base-peak value of a selected reference ion, normalized by the initial biomass of each sample as well by the internal standard concentration (2,3,3,3-d_4_-alanine). Samples were prepared in triplicates from two identical bioreactors for each strain.

## Results

Based on the hypothesis that the overproduction of phosphoglucomutase and transaldolase could help bypass the assumed bottleneck (Panagiotou et al., 2005c) in the metabolism of *F. oxysporum*, we previously described the expression of the *pgm* and *tal* genes under the regulation of the constitutive promoter of *gpd*A of *Aspergillus nidulans*. Strain FF11, which constitutively produced both enzymes, was selected for further evaluation. We reported how the overproduction of these two enzymes affected the metabolic channeling and the growth of the microorganism under aerobic conditions. A higher specific growth rate and a higher abundance for the majority of the detected amino and non-amino organic acids was observed at the end of the aerobic phase—when glucose was fully consumed or growth reached a plateau (Anasontzis et al., 2014b).

Considering the perspective of using *F. oxysporum* for ethanol production from lignocellulose in a consolidated bioprocess, we evaluated the effect of constitutive expression of the respective genes on ethanol productivity using glucose as the sole carbon source.

**Figure 1:**
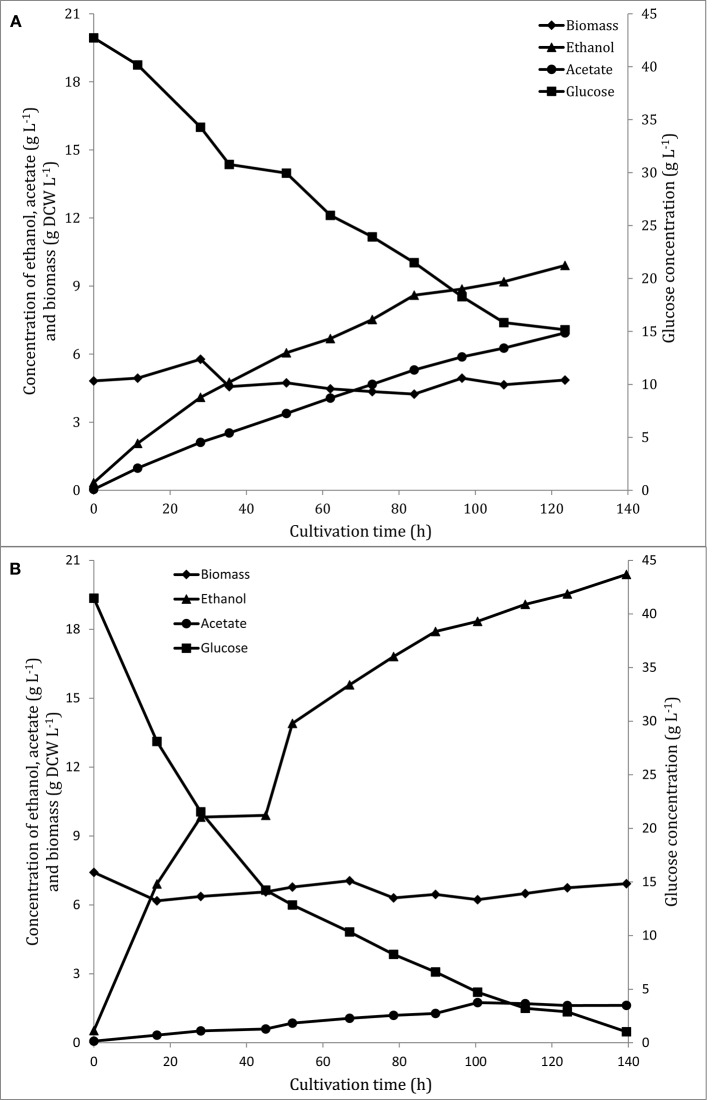
**Glucose (■), ethanol (▴), acetic acid (•), and biomass (♦) during the anaerobic phase of *F. oxysporum* F3 (A) and FF11 (B) fermentation**. These are results of duplicate measurements (technical duplicates) from two different fermenters (biological duplicates). Standard deviation was lower than 3%.

The recombinant strain, FF11, showed significantly higher ethanol yield than the wild type, and in parallel, a lower yield of acetic acid (Table 1). FF11 produced more than 20 g^.^L^−1^ of ethanol after 139 h of anaerobic fermentation, while the wild type seemed to decrease its glucose consumption rate at around 120 h, with an ethanol concentration of less than 10 g L^−1^. Another interesting point was the lower production of acetate by the recombinant strain compared to the wild type one; acetate is a common by-product of fermentation, and was also detected as a major by-product in the case of the wild type (Figure 1). Ethanol productivity was also higher for FF11 compared to F3 (0.146 and 0.080 g L^−1^ h^−1^, respectively), as was the glucose consumption rate (0.290 and 0.223 g L^−1^ h^−1^).

**Table 1:** **Yield of ethanol and acetic acid**.

	**F3**	**FF11**
**g PER g OF GLUCOSE CONSUMED**		
YSE	0.360	0.504
YSAc	0.252	0.040
Per cent EtOH	69.9%	98.1%
**g PER g OF INITIAL GLUCOSE**		
YSE	0.232	0.492
YSAc	0.162	0.039
Per cent EtOH	45.1%	95.7%

YSE, ethanol yield over consumed and initial glucose; YSAc, acetic acid yield over consumed and initial glucose; Per cent EtOH, percentage of ethanol over the theoretical yield of 0.51 g ethanol per g of glucose.

**Figure 2:**
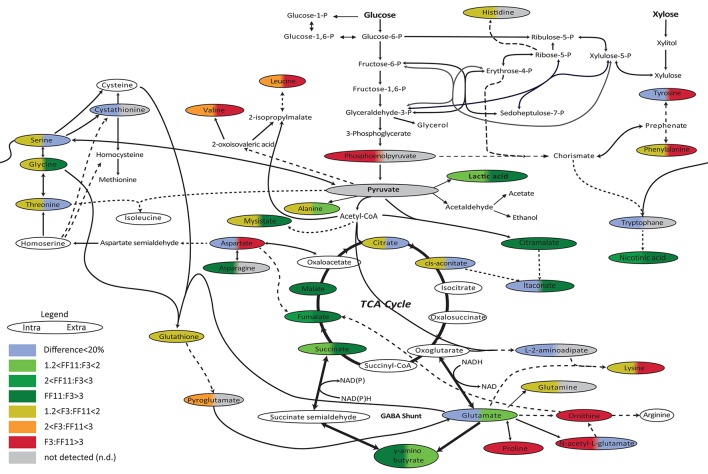
**Schematic demonstration of the relative intracellular concentrations of amino and non-amino organic acids during anaerobic growth of *F. oxysporum* F3 and FF11 strains on glucose**. Single lines: One enzyme reaction; dashed lines: several reactions; Colors on the left and right correspond to the difference in intracellular and extracellular metabolites, respectively. Red: F3:FF11 > 3; Orange: 2 < F3:FF11 < 3; green/yellow: 1.2 < F3:FF11 < 2: Dark green: FF11:F3 > 3; Green: 2 < FF11:F3 < 3; Light green: 1.2 < FF11:F3 < 2: blue: difference smaller than 20%; gray: not detected (adapted from Anasontzis et al., 2014a).

In order to further investigate the metabolic state of the two strains during the anaerobic stage of the culture, to identify possible bottlenecks, and to understand if the more efficient redox balance achieved by the recombinant strain during the aerobic phase was retained also under anaerobic conditions, we performed metabolomics analysis of amino and non-amino acid metabolites at the final sampling point of each fermentation—when glucose consumption reached a plateau for strain F3 and when glucose was practically depleted for strain FF11. The sampling point was selected in order to obtain comparable results with our previous work, when metabolomics analysis was performed at the end of the aerobic phase, and to allow accumulation of metabolites in detectable and significant levels (Anasontzis et al., 2014a). The results of the comparison between the two strains under anaerobic conditions are presented in Figure 2. It is evident that the citric acid cycle was more active in the recombinant strain, at least after the γ-aminobutyric acid (GABA) shunt. Higher concentrations of lactic acid, nicotinic acid, and citramalate were also observed. Itaconic acid levels were higher for the recombinant strain only in the extracellular samples, although still low as absolute concentrations. Several amino acids had lower concentrations in FF11 compared to the wild type strain. Table 2 shows the average values of the intracellular and extracellular metabolites under anaerobic conditions for both strains.

**Table 2:** **Amino acid and non-amino acid intracellular and extracellular metabolites detected at the end of the anaerobic phase, for the two strains**.

	**Anaerobic**			
	**Intracellular**		**Extracellular**	
	**F3**	**FF11**	**F3**	**FF11**
4-amino-n-butyrate (GABA)	1.0	66.1	7.4	10.1
Alanine	296.9	163.6	84.4	102.9
Asparagine	13.5	52.3	0.0	0.0
Aspartate	56.4	65.5	3.2	1.0
Cis-aconitate	19.0	12.3	0.3	0.3
Citramalate	0.0	0.6	0.6	3.8
Citrate	268.6	150.9	3.9	3.5
Cystathionine	1.0	1.0	0.0	0.0
Fumarate	18.2	45.7	2.2	17.3
Glutamate	75.4	71.7	1.8	2.4
Glutamine	3.3	1.9	0.0	0.0
Glutathione	9.6	6.6	0.6	0.3
Glycine	235.7	163.6	5.9	26.2
Histidine	19.8	14.1	0.0	0.0
Itaconate	8.3	7.2	0.0	0.6
L-2-amino-adipate	0.0	0.0	0.0	0.0
Lactic acid	41.5	82.5	9.3	84.2
Leucine	97.0	46.3	8.5	0.5
Lysine	43.6	26.4	1.1	0.1
Malate	17.9	76.6	1.3	11.6
Myristate	3.7	3.1	0.3	1.6
N-acetyl-L-glutamate	10.2	0.5	0.3	0.3
Nicotinate	1.7	4.1	0.0	0.2
Ornithine	73.9	8.8	2.2	0.7
Phenylalanine	24.8	15.8	2.1	0.5
Phosphenolpyruvate	1.1	0.0	0.0	0.0
Proline	279.5	14.3	31.8	4.8
Pyroglutamate	82.5	31.3	0.0	0.0
Pyruvate	n.d.	n.d.	0.0	0.0
Serine	88.9	57.7	0.2	0.2
Succinate	39.3	58.8	15.3	66.6
Threonine	88.9	57.7	0.8	0.7
Tryptophan	23.3	20.5	0.0	0.0
Tyrosine	25.7	27.8	1.2	0.2
Valine	150.3	63.3	7.4	1.1

All values express peak areas normalized by the biomass concentration. Standard deviation lower than 5%.

## Discussion

### Ethanol productivity

Our results show an increase in ethanol productivity in the recombinant strain. This indicates that the genetic modification performed was in the right direction, as it has improved the yield of the main product while reducing that of acetic acid, the main by-product. Phosphoglucomutase overexpression would be expected to increase the channeling of glucose to pyruvate and then to ethanol and acetic acid. However, it is not clear why acetaldehyde catabolism shifts toward ethanol instead of acetic acid. An explanation could be that due to the constitutive production of transaldolase and the more active PPP, the NADPH recycling is more efficient and therefore less acetic acid production is required to regenerate NADPH through the NADP-dependent acetaldehyde dehydrogenase (E.C. 1.2.1.3). The higher concentration of lactic acid in the recombinant strain confirms this, as lactic acid production regenerates NAD^+^ through the action of lactic acid dehydrogenase.

Despite the very high ethanol yield and the improvement in the ethanol productivity (Table 1), *F. oxysporum* still cannot compete with other microorganisms in terms of productivity and final ethanol concentration. As Table 3 shows, both these values from strains F3 and FF11 are still much lower in comparison to values in the literature. For example, *Rhizopus oryzae* has been reported to have an ethanol productivity of 1.87 g L^−1^ h^−1^ (Millati et al., 2005). The maximum ethanol concentration was also very low compared to values achieved routinely with *S. cerevisiae* in pure or mixed substrates (Saitoh et al., 2010). Nevertheless, FF11 gave among the highest yields of glucose to ethanol, higher than 98% of the theoretical value. In a similar study, Fan et al. demonstrated the effect of the expression of a transaldolase gene from *P. stipitis* in ethanol production in flask cultures (Fan et al., 2011a). Although the experimental set-up was different, and parameters such as productivity and yield are not readily comparable, extrapolation of their results indicates an improvement in ethanol yields. We observed even greater improvement, which could be attributed to simultaneous overproduction of both transaldolase and phosphoglucomutase.

**Table 3:** **Ethanol productivity of different strains reported in the literature, using glucose as the carbon source**.

**Strain**	**Ethanol concentration (g L^−1^)**	**Yield (g ethanol per g of sugars consumed)**	**Ethanol productivity (g L^−1.^h^−1^)**	**References**
*F. oxysporum* F3	9.9	0.36	0.081	This study
*F. oxysporum* F11	20.4	0.504	0.146	This study
*F. oxysporum* M209040-Tal2	8.5	~0.425	0.142	Fan et al., 2011a
*F. oxysporum CCTCC M209040*	7.5	~0.375	0.125	Fan et al., 2011a
*Mucor indicus*		0.46		Sues et al., 2005
*Saccharomyces* 1400 (pLNH33)	~110	0.4845	2.4	Krishnan et al., 1999
*Pichia stipitis* P5-90-133-p5-200-16*/Saccharomyces cerevisiae* V30		0.35–0.45		Kordowska-Wiater and Targonski, 2002
*Zymomonas mobilis* MTCC 92	70.4	0.495	2	Kesava et al., 1995
*Saccharomyces cerevisiae*		0.428–0.459		Shafiei et al., 2010
*Peniophora cinerea*	8.1	0.41	0.021	Okamoto et al., 2010
*Trametes suaveolens*	7.7	0.39	0.017	Okamoto et al., 2010
*Rhizopus oryzae* A	20.5	0.41	1.87	Millati et al., 2005
*Mucor corticolous*	21.5	0.43	1.48	Millati et al., 2005
*Mucor hiemalis*	19.5	0.39	1.44	Millati et al., 2005
*Mucor indicus*	19.5	0.39	1.41	Millati et al., 2005
*Saccharomyces cerevisiae*	21	0.42	1.29	Millati et al., 2005

### Metabolite concentrations

The relative levels of tryptophan, tyrosine, phenylalanine, and nicotinic acid for FF11, between the aerobic phase (Anasontzis et al., 2014a) and the anaerobic phase, showed no significant variation. For the wild type, though, all three of these amino acids showed higher levels in the anaerobic phase than in the aerobic one. It seems that in the wild type, their production is induced under anaerobic conditions. However, in the recombinant strain, there appears to be a constant need for their synthesis, indicating that the modifications have increased the metabolic flow toward their production in both culture regimes. The lack of detection of phosphoenolpuryvate under anaerobic conditions supports this idea, and it suggests increased channeling toward either the TCA cycle or the chorismate pathway. Although nicotinic acid levels are constant in the recombinant strain, they are lower in the wild type under anaerobic conditions.

For *S. cerevisiae*, it has been reported that under aerobic conditions nicotinic acid is synthesized from tryptophan, while under anaerobic conditions it appears to be produced from aspartate and glutamate (Ahmad and Moat, 1966). Strain F3 concentrations of nicotinic acid, glutamate, and aspartate drop during the anaerobic phase, an observation that indicates a similar mechanism of regulation. However, for the recombinant strain, although aspartate and glutamate also decrease under anaerobic conditions, nicotinic acid is only slightly affected. It is possible that the constitutive expression of the two genes has led to the partial retainment of the aerobic nicotinic acid production pathway (the chorismate pathway). Nicotinic acid is a precursor of NAD synthesis; however, it is not clear if the higher concentrations of nicotinic acid in the recombinant strain are related to the availability of the different forms of NAD.

The levels of all following amino acids—serine, glycine, alanine, leucine, threonine, lysine, glutamine, and proline—increased in the recombinant strain under aerobic conditions (Anasontzis et al., 2014a), but were relatively lower under anaerobic conditions. This could be loosely related to the protein production levels under the latter conditions. If the excessive production of fumarate during both phases in the recombinant strain acts as the electron acceptor during protein production, as has been previously suggested for *S. cerevisiae* (Liu et al., 2015), the intracellular concentrations of at least several of the protein building blocks could be expected to be lower, as they are more readily consumed. Nevertheless, under aerobic conditions the recombinant strain has higher intracellular levels of these amino acids, but whether this is a result of an increase in their production rate or a decrease in their consumption rate remains unclear with the current experimental set-up. The different relative levels of other amino acids such as valine, aspartate, asparagine, histidine, and glutamate further complicate the picture.

Apart from fumarate, other metabolites of the TCA cycle that are linked to the GABA shunt showed higher concentrations in the recombinant strain under both aerobic and anaerobic conditions. It seems that this effect is restricted after the GABA shunt and it is not reflected on the glutamate—the precursor of GABA—, citrate, and *cis*-aconitate levels. On the other hand, the GABA shunt of the wild type appears to be completely deactivated under anaerobic conditions, while the TCA cycle remains active, albeit with reduced metabolite concentrations. It seems that under anaerobic conditions, there is a reduced need in the wild type for GABA production, a compound that plays an important role in response to environmental stresses and especially to reactive oxygen stress (Mead et al., 2013), while the genetic modifications keep the GABA shunt active. The low production of acetic acid, which indicates no need for NADPH regeneration, may be associated with the active GABA shunt—as such pathways become extremely important as sinks for excess NAD(P)H which occurs when the TCA cycle is inhibited (Streeter and Salminen, 1990).

An active TCA cycle under anaerobic conditions has also been reported in other microorganisms. In *Rhizopus arrhizus*, it has been suggested that both TCA and pyruvate carboxylation are active but in the opposite direction (Kenealy et al., 1986; Roa Engel et al., 2008). The higher concentrations of fumarate, succinate, malate and GABA in the recombinant strain FF11 propose a similar equilibrium, leading to the accumulation of GABA. The higher succinate production could contribute to the NAD^+^ recycling, as it has been reported for *Bifidobacterium* sp. (Van der Meulen et al., 2006).

In order for the cell to regenerate its reductive potential, it is necessary to recycle the cofactors and maintain the balance of NAD^+^/NADH and NADP^+^/NADPH. This balance allows energy production and use of the substrate for compound synthesis (Roger et al., 2004). Both glycolysis and pentose phosphate pathway are two key metabolic pathways both present in even between distantly related microorganisms such as *F. oxysporum* (Panagiotou et al., 2005a) and lactic acid bacteria (Roger et al., 2004). Modification of the metabolic fluxes to increase or reduce the accumulation of a specific metabolite is often the target, but selection of the modification that will give the expected result is not always possible, as the effect can be unpredictable (Roger et al., 2004). The repression of an enzyme, such as the lactic acid dehydrogenase which also regenerates NAD^+^ (Monnet et al., 2000) or the acetaldehyde dehydrogenase which regenerates NAD(P)H (Panagiotou et al., 2005b), could drive the metabolism to alternative pathways and equilibria with the same or unexpected results.

The overproduction of phosphoglucomutase and transaldolase was not aimed at disrupting the redox equilibrium of *F. oxysporum*, but instead to accelerate the metabolism at specific points, and eventually increase its ethanol productivity. Nevertheless, it seems that the redox equilibrium has indeed been affected in a beneficial way, with several metabolites supporting it under both aerobic (Anasontzis et al., 2014a) and anaerobic conditions.

Despite the positive effect of the constitutive production of phosphoglucomutase and transaldolase directly on ethanol productivity and indirectly on redox equilibrium, further improvements would be required to allow the large scale perspectives of *F. oxysporum* in biofuel production from lignocellulose. Other aspects, such as ethanol tolerance, also need to be addressed (Paschos et al., 2015). Screening for the selection of wild type strains that are more efficient ethanol producers and better understanding of the gene regulation and bottlenecks under ethanol producing conditions (Ali et al., 2012, 2013), further genetic modifications on selected pathways, evolutionary engineering, and novel bioprocessing set-ups, are necessary in order to achieve the production of ethanol above the limit of 5% (w/v) from lignocellulose (Anasontzis and Christakopoulos, 2014). Under the scope of this work, investigation and increase of the efficiency of alcohol dehydrogenase would help determine other major bottlenecks in ethanol production from *F. oxysporum*, while studies of the performance of the selected strain on lignocellulose material would also shed light on the limitations that might have been removed or added through this approach. Constitutive production of these enzymes might not be the most optimal modification; thus fine-tuning using different promoters could improve the growth and ethanol characteristics of the modified strains.

## Author contributions

GA contributed to the design of the work, performed the fermentations and the sampling for metabolome analysis, interpreted the data, and drafted the manuscript. EK performed the fermentations, sampling, and HPLC analyses, and also drafted the results. SV designed and performed the metabolomics analysis and revised the manuscript. DH contributed to the design of the study, interpretation of the data, and revision of the manuscript. PC conceived the study, contributed to the design and interpretation of the results, and revised the manuscript. All the authors have approved the final version to be published and have agreed to be accountable for all aspects of the work.

## Funding

This work has been co-funded by the project PENED 2003 and the European Community's Seventh Framework Program (FP7/2007-2013) under grant agreement 213139 (the HYPE project). PENED is co-financed 80% from public expenditure through the EC-European Social Fund, 20% from public expenditure through the Greek Ministry of Development—General Secretariat of Research and Technology, and through private sector, under measure 8.3 of the OPERATIONAL PROGRAMME “COMPETITIVENESS” in the Third Community Support Program.

### Conflict of interest statement

The authors declare that the research was conducted in the absence of any commercial or financial relationships that could be construed as a potential conflict of interest.
